# The development of a decision support tool in the prehospital setting for acute chest pain – a study protocol for an observational study (BRIAN2)

**DOI:** 10.1186/s13049-024-01314-x

**Published:** 2025-01-06

**Authors:** Elin Lökholm, Carl Magnusson, Johan Herlitz, Annica Ravn-Fischer, Ola Hammarsten, Magnus Johansson, Kristoffer Hallin, Kristoffer Wibring

**Affiliations:** 1 Department of Prehospital Emergency Care, Sahlgrenska University Hospital, Gothenburg, Sweden; 2 Department of Molecular and Clinical Medicine, Institute of Medicine, Sahlgrenska Academy, University of Gothenburg, Gothenburg, Sweden; 3 PreHospen-Centre for Prehospital Research, Faculty of Caring Science, Work Life and Social Welfare, University of Borås, Borås, Sweden; 4 Department of Cardiology, Sahlgrenska University Hospital, Gothenburg, Sweden; 5 Department of Clinical Chemistry, Sahlgrenska University Hospital, Gothenburg, Sweden; 6 Ambulance Services, NU-sjukvården, Trollhättan, Sweden; 7 Ambulance Services, Skaraborg Hospital, Skara, Sweden; 8 Institute of Health and Care Sciences, Sahlgrenska Academy, University of Gothenburg, Gothenburg, Sweden; 9 Department of Ambulance and Prehospital Care, Region Halland, Sweden; 10 Varlabergsvägen 29, Kungsbacka, 434 39 Sweden

**Keywords:** Chest pain, EMS, Troponin, Prehospital, AMI, Emergency medical services, Ambulance

## Abstract

**Introduction:**

Chest pain is one of the most common reasons for contacting the emergency medical services (EMS). It is difficult for EMS personnel to distinguish between patients suffering from a high-risk condition in need of prompt hospital care and patients suitable for non-conveyance. A vast majority of patients with chest pain are therefore transported to the emergency department (ED) for further investigation even if hospital care is not necessary. Improved prehospital assessment and risk stratification, thus accurately and safely identifying patients suitable for non-conveyance, could prevent unnecessary transport to the ED. This would reduce ED crowding and overburdening sparse EMS resources. It would thus also probably reduce healthcare costs. Little is known about the prehospital use of the 5th generation, i.e. high-sensitivity troponin analyses. The aim of this project is to develop an EMS decision support tool using high-sensitivity troponin I for risk assessment of chest pain patients.

**Methods and analysis:**

This is a prospective, multicentre, cohort study including adult unselected EMS patients with chest pain. Data is being collected from 20 May 2023 to 31 December 2025, aiming to include at least 2,000 patients. High-sensitivity troponin I is being analysed bedside using Siemens Healthineers Atellica VTLi. In addition to prehospital troponin I, data is being collected on patient medical history, onset, vital signs, symptoms, ECG and diagnosis at hospital discharge. Several statistical analyses (random forest, logistic regression, gradient boosting) will be conducted to identify the best model for identifying patients with low-risk conditions suitable for non-conveyance.

**Ethics and dissemination:**

The study has been approved by the Swedish Ethical Review Authority (Dnr 2022-01066-01 and 2022-06846-02). Patients are being informed about the study both orally and in writing. The results of the study will be published in a peer-reviewed journal and will be presented at national and/or international conferences.

**Registration details:**

The study is registered at ClinicalTrials.gov (NCT05767619).

**Supplementary Information:**

The online version contains supplementary material available at 10.1186/s13049-024-01314-x.

## Strengths and limitations of this study

- Large prospective, multicentre study including unselected EMS patients with chest pain.
- Examining prehospital use of high-sensitivity troponin in real-life conditions.
- The EMS personnel is not being blinded to the troponin results, introducing the risk of bias in patient assessment and care.

**Supplementary Information:**

The online version contains supplementary material available at 10.1186/s13049-024-01314-x.

## Introduction

Emergency medical services (EMS) care has undergone dramatic changes in recent decades. Previously, the EMS focused on quick patient transport to the nearest emergency department (ED) with limited care and assessment on scene. Nowadays, EMS care is more focused on patient assessment and more extensive treatment on scene, thereby enabling non-conveyance or referral to a wide range of different destinations [1]. Therefore, “care at the right level of care” has become a catchphrase. Fast-tracks bypassing the ED, referral to primary care, and remaining on scene with self-care advice are now a natural part of EMS care, in addition to transport to the ED [2]. EMS care has become increasingly differentiated, not solely focusing on the prompt transport of critically ill patients to the nearest ED but also on handling patients with non-urgent care needs.

Chest pain is one of the most common reasons for contacting the EMS [3–5]. However, the above-described methods for differentiating EMS care are generally not available for patients with chest pain. Approximately 15% of EMS chest pain patients have a high-risk condition, for example acute myocardial infarction (AMI), pulmonary embolism or aortic dissection [6, 7]. Of these, fewer than a one-third have an ST-elevation myocardial infarction (STEMI) [6, 8], entitling them to fast-track transportation directly to percutaneous coronary intervention (PCI) centres for immediate revascularisation [9]. The remaining patients with a high-risk condition, suffering mainly from non-ST elevation myocardial infarction (NSTEMI), are transported to often crowded EDs [6]. In the ED, these patients receive further care and investigation along with the 2/3 of all EMS chest pain patients, which often in retrospect, are without need of hospital care, given their diagnosis at hospital discharge [6]. EMS differentiation between patients with time-sensitive conditions and patients without hospital care needs is thus largely deficient. It is difficult for EMS personnel to identify which patients are suffering from a high-risk condition requiring prompt hospital care and which patients are suitable for non-conveyance or referral to primary care. This is the reason why a vast majority of patients with chest pain are transported to the ED for further investigation even if hospital care is not necessary [10]. Furthermore, little is known about chest pain patients which today does not convey [6, 7, 11] and the accuracy of the EMS assessment [11].

In recent years, interest in prehospital analyses of troponins to improve prehospital patient assessment and risk stratification has increased [12–16]. However, these studies used 4th generation troponin analyses, and little is known about the prehospital use of 5th generation, i.e., high-sensitivity troponin analyses. Furthermore, previous studies have focused on the prehospital use of the HEART score even though the HEART score was developed on hospital data for hospital use and does not take high-risk diagnoses other than AMI into account [17]. In addition to prehospital analyses of troponins, the use of artificial intelligence (AI) algorithms [10, 18] and other statistical models has shown promising results regarding the identification of chest pain patients suitable for non-conveyance [17, 19].

Improved prehospital assessment and risk stratification, thus accurately and safely identifying patients suitable for non-conveyance, could prevent unnecessary transport to the ED. It would thus reduce ED crowding and overburdening sparse EMS resources [20]. It would probably also reduce healthcare costs [21, 22]. Furthermore, it would allow patients to avoid unnecessary EMS transport and long waiting times in the ED.

In the BRIAN 1 (BRöstsmärta In AmbulaNs (Swedish), EMS Chest Pain (English)) research programme [6, 17], we previously studied potential improvements in the EMS’ risk stratification of patients with chest pain. We have now started the data collection for BRIAN 2, in which we aim to develop an EMS decision support tool for the risk assessment of chest pain patients using AI and high-sensitivity troponin I. In BRIAN 2, we also aim to validate a previously developed decision support tool from BRIAN 1 [17] and the application of the European Society of Cardiology ESC 0/1 algorithm in the EMS setting [23]. This paper aims to present the study protocol and the initial feasibility of the study.

### Study objectives

To develop a decision support tool for risk stratification of patients with chest pain and validate previously developed decision support tools.

### Scientific questions

- To what extent will a developed decision support tool classify patients as low-risk and suitable for non-conveyance?
- What proportion of patients classified as low-risk will have a high-risk condition upon follow-up?
- Is a recently developed decision support tool ripe for inclusion in clinical routine?
- How does the decision support tool compare with existing algorithms and decision support tools for risk prediction in patients with chest pain?

## Methods and analysis

### Study design

Prospective multicentre observational study. Consecutive cases that meet inclusion criteria and lack exclusion criteria for a given period will be included in the study. The study is registered at ClinicalTrials.gov (NCT05767619).

### Population

Adult, unselected, EMS patients with a chief complaint of chest pain as assessed by EMS personnel.

### Inclusion criteria

- Chief complaint of chest pain or discomfort
- Primary EMS missions, i.e. not EMS transport from or between care facilities

### Exclusion criteria

- Age < 18 years
- Unwillingness to participate
- Inability to participate (language barriers, dementia, etc.)

### Setting

Data is being collected at four different sites. All EMS are tax-funded. Each ambulance is staffed by at least one ambulance nurse working with either another ambulance nurse or an emergency medical technician (EMT). Patients with STEMI on the prehospital Electrocardiogram (ECG) are transported directly to the nearest PCI centre, bypassing the ED or hospitals without PCI capabilities.

### Data collection sites

- EMS organisation in Göteborg, Lidköping and Uddevalla in Region Västra Götaland
- EMS organisation in Kungsbacka in Region Halland

### Kungsbacka

Kungsbacka is located in the northern part of Region Halland. The EMS organisation serves approximately 86,000 inhabitants and covers a catchment area of close to 1,500 km^2^. There are five ambulances during the day and three during the night, all manned by at least one registered nurse. There are approximately 600 primary EMS missions involving patients with chest pain annually. As part of routine care, a blood sample is collected during the EMS mission and brought to the ED along with the patient for high-sensitivity troponin T analysis at the hospital laboratory to speed up patient diagnostics at the ED. The results of the troponin analysis are not available to EMS personnel.

Most chest pain patients are transported to the closest regional hospital, located in Varberg, 52 km from Kungsbacka city centre. The hospital in Varberg lacks a PCI laboratory. Patients with STEMI or strongly deviating vital signs are transported to the Sahlgrenska University Hospital, located in Göteborg, 26 km from the Kungsbacka city centre. The Sahlgrenska University Hospital has a PCI laboratory running 24/7.

### Göteborg

Göteborg is the second-largest city in Sweden and is located in the western part of Västra Götaland County. The EMS organisation in Göteborg covers an area of 900 km², serving approximately 700,000 inhabitants. There are 16 ambulances operating during the daytime and 13 during the night, all manned by at least one registered nurse. The EMS system also provides two nurse-staffed single responders and one physician-staffed unit. There are three hospitals in Göteborg to which patients with chest pain are referred and transported, depending on their geographical location. Patients with STEMI or severely deviating vital signs are transported to the Sahlgrenska University Hospital, where it is possible to perform PCI 24/7, after contact with the hospital for a possible fast track/chain of care. There are approximately 45,000 primary EMS missions annually, of which 4,500 involve patients with chest pain.

### Lidköping

Lidköping municipality has an area of 1,400 km^2^ and approximately 40,000 inhabitants. A total of 24,000 of these inhabitants live in the city of Lidköping, which is the main city of Lidköping municipality. The closest hospital is in Skövde, approximately 50 km from Lidköping. The hospital in Skövde has both an ED and a PCI laboratory running 24/7. There are approximately 4,500 EMS missions annually, of which approximately 500 involve patients with chest pain.

### Uddevalla

Uddevalla is located approximately 90 km north of Göteborg. There are approximately 40,000 people living in Uddevalla city and 290,000 people living in the local EMS organisational catchment area. Most EMS patients are transported to NÄL hospital, located 30 km from Uddevalla, with an ED running 24/7. NÄL has a PCI laboratory running from Monday 8 am to Friday 4 pm. For the remaining time, patients with STEMI are referred to the Sahlgrenska University Hospital in Göteborg. There are approximately 4,000 primary EMS missions annually, of which 450 involve patients with chest pain.

### Data collection

Data is being collected from 20 May 2023 to 31 December 2025, aiming to include at least 2,000 patients.

Consecutive sampling is applied. When an EMS unit is assigned a mission concerning a patient with chest pain, the patient is asked by the EMS personnel if they want to participate in the study (informed consent). If so, data collection is initiated on site. The patients also obtain written information regarding the study, allowing them to inform themselves about the study under less stressful circumstances.

Data is being collected on several variables, including demographics, medical history, clinical presentation and symptoms, pain narrative, vital signs, ECG, and troponin, as well as follow-up data on hospital admission, diagnosis and mortality (Supplemental material 1). Variables have mainly been chosen based on the results of BRIAN 1 [6, 17] and other previous studies [8, 9].

Data is being collected using several different data sources. One is the study protocol, which is completed by EMS personnel during the EMS mission. The study protocol includes items regarding clinical presentation, pain narrative, ECG and troponin analyses. Depending on the study site, the study protocol is either integrated into the digital EMS medical records or completed on a separate digital platform (REDCap). Either way, both are filled in during the EMS mission using tablets.

From the EMS medical records, data is being collected regarding vital signs, EMS mission priority, triage and different EMS mission time stamps.

From the hospital medical records, data is being collected regarding age, sex, previous diseases, hospital contact within 72 h of EMS mission, ICD-10 diagnosis, hospital admission and mortality.

The ECG is obtained by the EMS personnel and digitally stored as part of the patient’s medical records. The EMS personnel interpret the ECG on site and state their findings in the study protocol. The ECG will also be interpreted retrospectively by ECG experts when the data collection has been concluded.

Data from the EMS and hospital records is being retrieved using automated data extraction from the data warehouses of the regions included.

Data from the different data sources is being linked together into a single data set used for data analyses using the patients’ personal security number and/or EMS mission event number (Table 1).

**Table 1:** Data collection variables

	Definition/description	Data source
**EMS mission data**		
Priority out (Emergency dispatch centre, EMD)	Priority 1-3, where 1 is highest, i.e. lights and sirens	EMS medical record
Priority in (Emergency medical services, EMS)	Priority 1-4, where 1 is highest, i.e. lights and sirens	EMS medical record
EMS missions created at EDM, time stamp		EMS medical record
EMS mission received from EMD, time stamp		EMS medical record
EMS mission acknowledged by EMS, time stamp		EMS medical record
EMS arrival on scene, time stamp		EMS medical record
EMS patient transport initiated, time stamp		EMS medical record
EMS arrival at destination, time stamp		EMS medical record
EMS return initiated, time stamp		EMS medical record
Destination for EMS transport	Type of care facility to which the patients were transported	EMS medical record
Triage colour according to EMS triage	Red, Orange, Yellow or Green	EMS medical record
Triage colour according initial vital signs	Red, Orange, Yellow or Green	EMS medical record
Triage colour according to patient signs and symptoms	Red, Orange, Yellow or Green	EMS medical record
**Demographics and medical history**		
Age	Years	Hospital medical record
Sex	Male/Female	Hospital medical record
Acute coronary syndrome (ACS)	Any of the following ICD-10 codes: I200, I21, I22, I24, I252 or I254	Hospital medical record
Chronic obstructive pulmonary disease (COPD)	Any of the following ICD-10 codes: J44	Hospital medical record
Angina pectoris	Any of the following ICD-10 codes: I201, I208 or I209	Hospital medical record
Hypertension	Any of the following ICD-10 codes: I10, I11, I12, I13 or I15	Hospital medical record
Diabetes mellitus	Any of the following ICD-10 codes: E10, E11 or E14	Hospital medical record
Heart failure	Any of the following ICD-10 codes: I110, I130, I132, I255, I311, I420, I421, I422, I424, I425, I426, I427, I429 or I50	Hospital medical record
Stroke (ischaemic)	Any of the following ICD-10 codes: I63, I64, I65 or I66	Hospital medical record
Renal disease	Any of the following ICD-10 codes: I12, I13, I701, N17, N18, N19, N280, N288 or N289	Hospital medical record
Rheumatism	Any of the following ICD-10 codes: M05 or M06	Hospital medical record
Cancer	Any of the following ICD-10 codes: C1-9	Hospital medical record
Atrial fibrillation/flutter	Any of the following ICD-10 codes: I48	Hospital medical record
Psychiatric diagnosis	Any of the following ICD-10 codes: F1-6	Hospital medical record
Hyperlipidemia	Any of the following ICD-10 codes: E78	Hospital medical record
Previous coronary artery bypass graft (CABG) operation	Any of the following ICD-10 or procedure codes: FNA, FNC, FNE or Z951	Hospital medical record
Previous percutaneous coronary intervention (PCI)	Any of the following ICD-10 or procedure codes: FNG or Z955	Hospital medical record
Smoking	Never smoked; Stopped smoking >1 months ago; Still smoking one month before EMS mission; Unknown	EMS study protocol
**Vital sign (first registered value during EMS mission)**		
Breathing rate		EMS record
Oxygen saturation		EMS record
Systolic blood pressure		EMS record
Diastolic blood pressure		EMS record
Heart rate		EMS record
Level of consciousness		EMS record
Body temperature		EMS record
B-glucose		EMS record
**Clinical presentation**		
Paleness	Based on EMS assessment	EMS study protocol
Clamminess	Based on EMS assessment	EMS study protocol
Nausea (and/or vomiting)	According to patient or witnesses	EMS study protocol
Pain onset initiating EMS contact > 3 h ago	According to patient or witnesses	EMS study protocol
Continuous pain ≥20 min	According to patient or witnesses	EMS study protocol
Pain onset in connection with physical activity	According to patient or witnesses	EMS study protocol
Sudden pain debut (seconds)	According to patient or witnesses	EMS study protocol
Palpation tenderness	According to patient	EMS study protocol
Pain affected by movement	According to patient	EMS study protocol
Pain affected by breathing	According to patient	EMS study protocol
Affected breathing	According to patient	EMS study protocol
*Pain quality*		
Pressuring/heaviness	According to patient	EMS study protocol
Stabbing	According to patient	EMS study protocol
Discomfort	According to patient	EMS study protocol
*Pain localisation*		
Central pain	According to patient	EMS study protocol
Left side of chest	According to patient	EMS study protocol
Right side of chest	According to patient	EMS study protocol
Lower part of chest	According to patient	EMS study protocol
*Size of area affected by pain*		
Two inch diameter	According to patient	EMS study protocol
Size of patient’s palm	According to patient	EMS study protocol
Entire chest	According to patient	EMS study protocol
*Pain radiation*		
Jaw/throat	According to patient	EMS study protocol
Left arm	According to patient	EMS study protocol
Right arm	According to patient	EMS study protocol
Back	According to patient	EMS study protocol
Stomach	According to patient	EMS study protocol
No other pain	According to patient	EMS study protocol
Pain intensity according to NRS/VAS	According to patient	EMS study protocol
*Verbal pain intensity scoring*	According to patient	
No pain		EMS study protocol
Mild pain		EMS study protocol
Moderate pain		EMS study protocol
Severe pain		EMS study protocol
**Electrocardiogram**,** ECG**		
*EMS ECG interpretation*		
Sinus Rhythm, SR	EMS assessment	EMS study protocol
Atrial Fibrillation/Flutter, AF	EMS assessment	EMS study protocol
ST-Elevation	EMS assessment	EMS study protocol
ST-Depression	EMS assessment	EMS study protocol
T-wave Inversion	EMS assessment	EMS study protocol
Normal ECG	EMS assessment	EMS study protocol
*ECG interpretation by expert in retrospect*		
Sinus Rhythm, SR	ECG expert assessment	Hospital medical record
Sinus Bradycardia	ECG expert assessment	Hospital medical record
Sinus Tachycardia	ECG expert assessment	Hospital medical record
Supraventricular Tachycardia, SVT	ECG expert assessment	Hospital medical record
Atrial Fibrillation/Flutter, AF	ECG expert assessment	Hospital medical record
Ventricular Tachycardia, VT	ECG expert assessment	Hospital medical record
Atrial Pacing	ECG expert assessment	Hospital medical record
Ventricular Pacing	ECG expert assessment	Hospital medical record
AV-block II type 1	ECG expert assessment	Hospital medical record
AV-block II type 2	ECG expert assessment	Hospital medical record
AV-Block III	ECG expert assessment	Hospital medical record
ST-Elevation	ECG expert assessment	Hospital medical record
ST-Depression	ECG expert assessment	Hospital medical record
T-wave Inversion	ECG expert assessment	Hospital medical record
Q-wave	ECG expert assessment	Hospital medical record
Premature Ventricular Contraction, PVC	ECG expert assessment	Hospital medical record
Premature Atrial Contractions, PAC	ECG expert assessment	Hospital medical record
Left Bundle Branch Block, LBBB	ECG expert assessment	Hospital medical record
Right Bundle Branch Block, RBBB	ECG expert assessment	Hospital medical record
Sinus rhythm and none of abnormalities above	ECG expert assessment	Hospital medical record
QRS-duration	ECG expert assessment	Hospital medical record
Prehospital Troponin I value	Analysed using Siemens Atellica VTLi	EMS study protocol
Time stamp for Troponin I value		EMS study protocol
Troponin T value	Analysed inhospital using Roche Cobas, modul e801, blood sample retrived during EMS missions	Hospital medical record
Time stamp for blood sample collection for Troponin T		Hospital medical record
ED visit within 72 h from EMS mission		Hospital medical record
Hospital admission to hospital ward		Hospital medical record
Diagnosis on hospital discharge, ED	ICD-10 code	Hospital medical record
Diagnosis on hospital discharge, Epicris	ICD-10 code	Hospital medical record
30-day mortality from EMS mission		Hospital medical record

### Outcome measures

#### Primary outcome

1. Proportion of patients classified as low-risk by the decision support tool.
2. The proportion of patients, among those classified by the decision support tool as low-risk, who at follow-up turn out to have a high-risk condition.

#### Secondary outcome

1. Proportion of patients classified by the decision support tool as low-risk in relation to sex and age.
2. The proportion of patients, among those classified by the decision support tool as low-risk in relation to sex and age, who at follow-up turn out to have a high-risk condition.
3. Proportion of patients among those who have been classified as low-risk and transported to an ED and who have been admitted to a ward (discrepancy between the assessment by the decision support tool and the assessment in the ED).
4. Distribution of final diagnoses in the above-mentioned group.
5. In retrospect, the predictive ability of the decision support tool regarding both low-risk and high-risk patients.

### Definitions

Risk condition classification is based on the diagnosis given at discharge from the hospital according to the physician in charge. A low-risk condition is defined as a diagnosis with no medical need for hospital treatment, suitable for non-conveyance. This also includes patients with chest pain who remain on scene, who do not visit the ED within 72 h and who do not die within 7 days.

A high-risk condition is defined as a time-sensitive condition with relatively high mortality rate in need of immediate medical care when seen retrospectively, for which transport to the hospital with highest priority is called upon. These definitions are the same as those used in BRIAN 1 [6].

An intermediate-risk condition is defined as a diagnosis probably in need of hospital care but for which time is not judged to be a critical factor. That is, it does not fulfil either of the definitions above.

### Statistical analyses

Descriptive data will be presented as mean and standard deviation or median and interquartile range for numeric variables, while numbers and percentages will be reported for categorical variables. Univariable analysis will be conducted using logistic regression, adjusted for age, and odds ratios with 95% confidence intervals will be presented.

A multivariable prediction model for acute chest pain will be developed using multivariable logistic regression with stepwise selection, LASSO logistic regression, two-stage LASSO, and adaptive LASSO. Additionally, machine learning models using classification trees, gradient boosting trees, and/or random forests will be applied. Hyperparameter tuning for the LASSO and machine learning methods will be conducted to minimise the model’s using cross-validation deviance. Model performance will be evaluated using pseudo-R-square for overall performance, along with receiver operating characteristic (ROC) curves and area under the curve (AUC) for discriminatory assessment. Bootstrapping will be used for internal validation. Calibration will be assessed using calibration-in-the-large, calibration slope, and calibration plots. Variable importance of the included predictors will be evaluated by multivariable R-square decomposition using Shapley values. Missing data will be handled using multivariate imputation by chained equations to capture complex nonlinear relationships and interactions in the data.

Subgroup analyses will focus on the additional clinical utility of prehospital Troponin I in high-risk patients not identified as such by the current EMS triage system/guidelines — specifically, patients transported to the emergency department (ED) without obvious signs of a time-sensitive condition. Additionally, analyses will be considered by age and sex to determine in where the model demonstrates the highest clinical utility.

All statistical tests will be conducted at the 5% significance level. Statistical analyses will be conducted using the R language and environment for statistical computing (R Core Team, Vienna, Austria) and the Python programming language (Python Software Foundation, Delaware, United States).

### Sample size

Sample size considerations were based on the three criteria of Riley et al. [27]:

i) small overfitting, defined by an expected shrinkage of predictor effects by 10% or less,
ii) small absolute difference of 0.05 in the model’s apparent and adjusted Nagelkerke’s R-squared value, and.
iii) precise estimation (within +/-5% points) of the average outcome risk in the population.

Assuming an event rate of 68%, an AUC of 0.85 [9] and considering 80 evaluated parameters (counting categorical variables as one parameter per degree of freedom), 1857 patients are needed in total. To be cautious and to compensate for missing data, 2,000 individuals will be included.

Calculations were performed by using the R language and environment for statistical computing (R Core Team, Vienna, Austria) version 4.2.3 with the pmsampsize package version 1.1.3.

### Patient and public involvement

None.

### Prehospital troponin analysis

Troponin I is being analysed during EMS missions using Siemens Healthineers Atellica VTLi patient side immunoassay analyser. Atellica VTLi is a point of care (POC) high-sensitivity troponin I assay with a level of detection (LoD) of 1.2 ng/L in plasma and 1.5 ng/L in whole blood, the limit of quantification based on a 20% coefficient of variation of 2.1 ng/L for plasma and 3.7 ng/L for whole blood. The sex-specific 99th percentile URLs were reported to be 27 ng/L for male patients and 18 ng/L for female patients [24–26]. Troponin is being analysed using either venous lithium heparinised whole blood or capillary whole blood depending on the study site and/or patient/personnel preferences.

The Atellica VTLi instruments are tested once each month by study representatives according to the manufacturer’s instructions.

Fifteen instruments are used for troponin I analyses. The instruments are stored at the EMS station in docking stations for battery charging. When EMS personnel are starting their shifts, an instrument is placed in the ambulance along with necessary blood sampling equipment. When caring for a patient with chest pain, the instrument is brought to the patient, and troponin is analysed bedside after retrieving informed consent for study participation. The Atellica VTLi must be in a horizontal position and stationary during the analysis process. Therefore, the analysis must be completed before initiating patient transport.

The analysis process takes about 8 min. The EMS personnel are instructed to initiate the troponin analyses as quickly as possible to minimise delay. Before the start of the study, the median time on the scene was approximately 25 min. This time frame allows the 8-minute analysis to be executed with minimal delay if initiated quickly after scene arrival. The EMS personnel are carefully instructed not to allow the results of the troponin analysis to affect clinical care. Most importantly, they should not recommend non-conveyance based on the troponin value achieved. Patients should, in all cases, be cared for only in line with organisational guidelines.

The EMS personnel are trained by Siemens Healthineers and/or study representatives on how to operate the Atellica VTLi. Study representatives are also available through the data collection period for further guidance and support.

Atellica VTLi cartridges for troponin I analyses require cold storage (+ 2-+8 °C). Cartridges can only be stored at room temperature for 24 h. Cold storage is not standard equipment in the ambulances of the current study sites. The need for cold storage is being handled in different ways for different study sites. Cartridges at densely populated sites with many EMS missions per shift are being kept at room temperature during the shift. If not used during the shift, the cartridges are jettisoned. For study sites with fewer EMS missions per shift, mini refrigerators, made for insulin travel storage, are installed in ambulances. These are powered by 5 V USB-A ports, 3.4 A, 10 W. Each mini refrigerator can store two cartridges.

### Ethics and dissemination

The study has been approved by the Swedish ethical review authority (Dnr 2022-01066-01 and 2022-06846-02). The study is observational, and there is no patient intervention other than retrieving blood samples. Patients are informed about the study both orally and in writing. Consent to participate in the study is confirmed in writing. The results of the study will be published in a peer-reviewed journal and will be presented at national and/or international conferences.

Data is being stored under pseudonyms making patient identification impossible without access to the hospital or EMS journal systems. Data is being stored using the data security and protection solutions of the regions included to minimise the risk for external data access.

### Present status and discussion

By December 2023, 380 EMS missions have been included. This is lower than expected. Inclusion has been hampered by malfunctioning of two of the instruments. One of them was due to reckless handling, and the other was due to the presentation of repeated error messages. Patient inclusion varies substantially between different study sites and between different EMS personnel. This variability indicates that the main reason for not including patients is a lack of compliance by EMS personnel, i.e., not recruiting eligible patients for inclusion. However, the inclusion rate has successively increased over time.

In approximately 15% of analyses with the Atellica VTLi, there is an error message, or the instrument is not able to provide a result. Early in the data collection period, unsuccessful troponin analyses were more common, which can probably be explained by instrument unfamiliarity and lack of training. In general, the Atellica VTLi is considered easy to use and functions well in the EMS setting. However, it should be taken into account that it is not always possible to obtain a troponin analysis result.

The process of obtaining informed consent in the EMS setting has previously been described as challenging [27, 28]. So far, the process of obtaining informed consent has not been an issue. The EMS personnel involved describe the process as easy, and few patients refuse to participate. The main challenge is instead to motivate EMS personnel to include eligible patients.

Situations concerning patients with a highly elevated troponin levels have been identified by the EMS personnel as difficult to handle. Personnel have been instructed to care for all patients in line with current guidelines. However, the EMS personnel describe a moral obligation to act upon elevated troponin value, by for example increasing pace of care or informing the receiving care unit regarding the elevated troponin levels.

As EMS personnel are not being blinded to the troponin value, the research team is fearful that EMS personnel may act on the results of the troponin analysis, by referring the patient to non-conveyance in the case of low troponin levels. They would thereby be deviating from current guidelines and potentially putting patients at risk by delaying care despite time-sensitive conditions.

A major challenge in the EMS setting is the need of keeping the Atellica VTLi cartridges cooled, despite the robustness and reliability of the mini refrigerators used. Substantial numbers of cartridges are jettisoned on a regular basis, resulting in increasing costs.

### Interim safety analysis

An interim analysis was conducted after the inclusion of the first 100 patients to ensure patient safety during the initial phase of the study. The primary objective of this interim analysis was to verify that no patients were non-conveyed based solely on troponin readings. This analysis served as an a priori safety check to confirm that EMS decisions regarding patient transport were based on a comprehensive clinical assessment rather than troponin results alone. Upon arrival at the scene, the EMS followed a standardised clinical guideline that includes a thorough evaluation of the patient’s clinical presentation, medical history, vital signs and ECG findings.

During the interim analysis, detailed data from 141 patients was reviewed to assess the EMS’ decision-making process. Each case was evaluated to determine the rationale behind the conveyance decision. Specifically, the analysis focused on identifying instances in which the troponin reading could have disproportionately influenced the decision not to transport a patient. Patient outcomes were tracked to identify any adverse events according to the definition or need for subsequent medical attention after a non-conveyance decision. This step ensured that patients who were not transported did not experience adverse outcomes that could have been prevented or mitigated by hospital transport.

Among the 141 patients, a total of 21 patients (14.9%) were not conveyed after assessment by the EMS. The rate of non-conveyance is thus similar to, and even lower than, that of the patient population with chest pain assessed within the EMS organisations (approximately 20% non-conveyance rate). In accordance with the study outcome, we encourage EMS personnel to obtain a full history of the patient care event, including physician diagnosis at hospital discharge, which may explain the increased conveyance rate. Moreover, since the troponin analysis results are not being blinded, elevated troponin levels could result in transporting patients to the ED also in the case of patients who would normally be non-conveyed.

Of the patients who were non-conveyed and referred to lower levels of care, seven patients (33%) sought emergency care and were admitted to the ED within 72 h. An evaluation of patient outcomes revealed that none of the non-conveyed patients fell into the high-risk category. All but one were classified as low-risk. This single patient in the intermediate-risk category was classified based on the final diagnosis, in accordance with the study definitions previously described by Wibring et al. [6].

### Rationale for the decision support tool

The decision support tool will be created based on the information given from four different aspects of the clinical assessment: 1) Patient demography (age, sex and previous history, 2) Acute symptoms and clinical findings, 3) ECG findings and 4) Troponin analyses. The relative contribution of each of these four aspects is not known a priori and will most likely differ between cases.

## Conclusions

Using the Atellica VTLi in the EMS setting for the analysis of troponin I in acute chest pain patients is feasible. However, the instruments are unfamiliar for most EMS personnel, and a break-in period should be allowed for when introducing this kind of equipment in the EMS setting.

The need to keep the Atellica VTLi cartridges cooled is difficult to handle in the EMS setting. Prolonged room temperature storage ability of Atellica VTLi cartridges would substantially improve EMS feasibility and reduce costs.

EMS personnel not recruiting eligible patients for study inclusion due to lack of compliance may prolong data collection. Motivating personnel to include patients is a cornerstone for study success.

## Electronic supplementary material

Below is the link to the electronic supplementary material.

Supplementary Material 1

## Data Availability

No datasets were generated or analysed during the current study.
