# Supplementary material for: The development of a decision support tool in the prehospital setting for acute chest pain – a study protocol for an observational study (BRIAN2)
Source: Scand J Trauma Resusc Emerg Med. 2025 Jan 6;33:4. doi: 10.1186/s13049-024-01314-x (PMC11706110; doi:10.1186/s13049-024-01314-x)
Supplement: Supplementary file 1 — Supplementary Material 1 [file 13049_2024_1314_MOESM1_ESM.docx]

# Information for research participants about participation in a research study

## What is the study about and why do you want me to participate?

We have interpreted your main complaint as pain/discomfort in the chest. This is one of the most common reasons for patients to contact the EMS. Discomfort or pain in the chest can be caused by a variety of conditions, ranging from serious conditions such as heart attack to more benign conditions such as muscle inflammation. Some cases require urgent care at a hospital, while more benign conditions do not need hospital care at all. Currently, it can sometimes be difficult for the EMS personnel to determine what is causing a patient's chest pain and how serious the patient's condition is. The current research study aims to develop a decision support tool that will, in the future, assist EMS personnel in making decisions regarding the further care of patients with chest discomfort. This is so that future patients with chest discomfort can receive improved care already in the ambulance, tailored to the severity of their condition.

## How the study is conducted

The decision support tool being developed is based on information about:

- Age, sex, and medical history.
- Symptom presentation and the circumstances under which the discomfort began.
- ECG and measurements taken by EMS personnel, such as pulse and blood pressure.
- Enzyme levels in the blood that indicate possible heart involvement (a blood sample taken either capillary through a finger stick or venously through an arm stick, analysed on-site by EMS personnel, which is otherwise always taken upon arrival at the hospital).
- Blood sugar control based on the above-mentioned blood sample.

Information is also collected from your medical records about:

- Continued care within the healthcare system (hospitalisation, emergency department visits).
- Diagnosis given as the cause of the chest discomfort.
- Survival.

Apart from the above-mentioned blood sample, these are routine data collected in connection with EMS care of patients with chest discomfort. The above data are retrieved by the research team from the questionnaire filled out by EMS personnel and the data collected during the ongoing EMS mission, which is registered digitally on a protected data server. To be able to obtain this information for the research study, we need your consent.

For the research participant, participation in the study means that information about previous illnesses, ECG, and measurements are retrieved from the region's database and the population register to obtain the information and compile it with the data registered digitally during the ongoing EMS mission. Your care and treatment will not be affected by your participation in the study, other than that EMS personnel will take a blood sample on-site (which is also routinely taken at the hospital for patients with chest discomfort and is discarded immediately after analysis). Therefore, the research participant does not need to do anything and will not be contacted by the research team in the future.

## Possible Benefits and Risks of Participating in the Study

There are no significant risks associated with participating in the study, as your participation will not affect your ongoing care. However, the blood sample taken by the EMS personnel may potentially expedite your care at the hospital if it indicates a heart attack.

## What happens to my data?

The purpose of processing your personal data is solely for this research project to develop a prehospital decision support tool for people with chest pain/discomfort. The legal basis according to the EU General Data Protection Regulation (GDPR) is that the processing of personal data is necessary to perform a task in the public interest.

The research team (consisting of researchers from Sahlgrenska University Hospital, the Västra Götaland Region, Region Halland, and the University of Borås) will compile the information about you and store it on a data server belonging to the Västra Götaland Region. Thus, the information will be protected by the Västra Götaland Region's IT security system. The data will be handled confidentially, meaning that the collected information will not be saved together with your name or personal identification number. Each person will instead be identified via a code that can only be linked to you as an individual if one has access to the region's medical record system. The information will be processed in such a way that unauthorised persons cannot access it. The data will be stored for at least 10 years in accordance with the Archive Act (SFS1990:782).

The data controller is the Västra Götaland Region. According to the EU General Data Protection Regulation, you have the right to access the information about you that is being processed in the project free of charge and, if necessary, have any errors corrected. You can also request that your data be deleted and that the processing of your personal data be restricted. However, the right to deletion and restriction of the processing of personal data does not apply when the data is necessary for the current research.

If you wish to access the information, please contact the principal investigator:

Carl Magnusson, Ambulans och prehospital akutsjukvård, Sahlgrenska Universitetssjukhuset, Adress XXXXXX. Phone number: XXXX-XXXXXX.

Data protection officers in each region can be reached as follows:

[dataskydd@regionhalland.se](mailto:dataskydd@regionhalland.se) (Region Halland)

[sahlgrenska.universitetssjukhuset.dso@vgregion.se](mailto:sahlgrenska.universitetssjukhuset.dso@vgregion.se) (Sahlgrenska Universitetssjukhuset)

[skas.dso@vgregion.se](mailto:skas.dso@vgregion.se) (Skaraborgs Sjukhus)

[nu.dso@vgregion.se](mailto:nu.dso@vgregion.se) (Norra Älvsborgssjukhus and Uddevalla sjukhus, NU-sjukvården)

The data collected about you within the framework of the research study will never be disclosed and will remain confidential and inaccessible to anyone other than those responsible for the study. The results of the statistical analyses that will be conducted to develop and quality assure the decision support tool will be presented at group level without identifying individual participants. The results will be published in scientific journals and presented at scientific conferences.

If you are dissatisfied with how your personal data is being handled, you have the right to file a complaint with the Swedish Authority for Privacy Protection (Integritetsskyddsmyndigheten), which is the supervisory authority.

## Insurance and compensation

Participation in the study does not provide any additional insurance coverage, but the research participants are insured through the respective healthcare provider's patient insurance via the mutual insurance company of the county councils, Löf. No compensation is provided for participating in the research project.

## Participation is voluntary

Your participation is completely voluntary, and you are free to withdraw from participation at any time, even after you have agreed to participate, without having to provide any reason for doing so. If you choose to withdraw, please contact the principal investigator or the contact person in the respective region as listed below. We are, of course, grateful if you choose to participate, but we also fully understand if you choose to decline for any reason.

If you choose to participate, we would be grateful if you sign the consent form.

## Responsible for the study

Research Principal Investigator

Västra Götalandsregionen

Principal Investigator

Carl Magnusson

Ambulans och prehospital akutsjukvård

Sahlgrenska Universitetssjukhuset

Gullbergstrandgata 36C, 411 04 Göteborg

Kontaktperson Västra Götalandsregionen

[carl.magnusson@vgregion.se](mailto:carl.magnusson@vgregion.se)

Kontaktperson Region Halland

[kristoffer.wibring@regionhalland.se](mailto:kristoffer.wibring@regionhalland.se)

Case number __________________________ (to be filled out by the EMS personnel)

## Consent to Participate in the Project

I have received verbal and/or written information about the study and have had the opportunity to ask questions. I will retain the written information.

I consent to participate in the project "Development of a Decision Support Tool for Optimizing Prehospital Assessment of Acute Chest Pain."

| Place and date | Signature |
| --- | --- |
|  |  |
|  | Clarification of Name |
|  |  |
